# Recommendations for improving primiparous women’s childbirth experience: results from a multiphase study in Iran

**DOI:** 10.1186/s12978-021-01196-7

**Published:** 2021-07-06

**Authors:** Solmaz Ghanbari-Homaie, Shahla Meedya, Sakineh Mohammad-Alizadeh-Charandabi, Mohammad Asghari Jafarabadi, Eesa Mohammadi, Mojgan Mirghafourvand

**Affiliations:** 1 Department of Midwifery, Faculty of Nursing and Midwifery, Tabriz University of Medical Sciences, Tabriz, Iran; 2 Member of South Asia Infant Feeding Research Network (SAIFRN), School of Nursing, Faculty of Science, Medicine and Health, University of Wollongong, New South Wales, Australia; 3 Social Determinants of Health Research Center, Tabriz University of Medical Sciences, Tabriz, Iran; 4 Road Traffic Injury Research Center, Tabriz University of Medical Sciences, Tabriz, Iran; 5 Department of Statistics and Epidemiology, Faculty of Health, Tabriz University of Medical Sciences, Tabriz, Iran; 6 Department of Nursing, School of Medicine, Tarbiat Modares University, Tehran, Iran

**Keywords:** Childbirth experience, Maternal satisfaction, Recommendation, Delphi technique

## Abstract

**Background:**

Women's satisfaction with childbirth experience is considered as one of the quality indicators of the maternity services across the world. However, there is no guideline for improving the experience of childbirth in Iran that is suitable for women with different cultural, economic, and social statuses. The aim of this study is to make recommendations for practice and propose a clinical guideline for improving the experience of women with vaginal births.

**Methods/design:**

The study design was a mixed method study with a sequential explanatory approach consisting of three phases. The first phase of the study was a cross-sectional study to identify the predictors of traumatic vaginal childbirth experience among 800 primiparous women from Tabriz health centers who had vaginal birth. Data collection tools in this phase were Childbirth Experience Questionnaire (CEQ) and Support and Control in Birth (SCIB). Both tools were validated for Farsi language. The second phase was a qualitative study with 17 in-depth individual interviews among women who took part in the first phase to better understand their reasons that influenced their childbirth experience either positively or negatively. The third phase of the study was to develop recommendations for a proposed clinical guideline through a Delphi study where maternal health experts were selected and invited to take part in the panel. They first rated the proposed recommendations individually and provided written responses on their own agreement or disagreement with each statement in terms of its impact on childbirth experience, feasibility, acceptability, and cost-effectiveness. After three confirmation rounds, the final conscience was reached by the panel members.

**Results:**

The results of the quantitative phase showed that the probability of negative experience of childbirth was increased when physical exercise was not implemented during pregnancy, lacking pain relief options, having fear of childbirth, lacking skin to skin contact with the newborn and being unable to initiate breastfeeding in the first hour after birth (*P* < 0.05). The analysis of qualitative data revealed 13 major theme categories which were related to women’s sense of internal control, external control and support. In the third phase of the study, culturally appropriate recommendations were made and an evidence-based clinical guideline was proposed. The proposed guideline was based on the combination of the quantitative and qualitative phases, a review of the literature, and the opinions of Iranian experts using the Delphi technique.

**Conclusion:**

Given the high prevalence of negative childbirth experience among Iranian primiparous women, the present study may be of great interest for managers, leaders, policymakers, and care providers to improve the quality of the maternity services. However, further studies are required to translate the recommendations into practice and identify enablers and barriers during the implementation of the proposed guideline. To adopt the recommendations at national level, there is a need to further studies to assess the effectiveness of the proposed guideline within different communities across the region and the country.

**Supplementary Information:**

The online version contains supplementary material available at 10.1186/s12978-021-01196-7.

## Introduction

Childbirth experience is one of the most important events in a woman's life [1]. Women clearly remember their childbirth experiences even after numerous years and often describe their childbirth experience as a positive or negative life experience [2]. Childbirth experiences can affect the personal and social life of a woman, her child and family [3]. For instance, a positive experience of childbirth can lead to advantageous outcomes such as high maternal self-esteem, and better neonatal growth and development [4, 5]. However, a negative childbirth can lead to disadvantageous outcomes such as fear of subsequent childbirth [6], postpartum depression [7], and poor breastfeeding outcomes [8]. A woman's satisfaction with her childbirth experience depends on several factors, such as feeling in control [9], receiving support [10], the intensity of pain experienced during labour [11], and power dynamics between the woman and the care provider [12].

Satisfaction with the childbirth experience is considered as one of the quality indicators of the maternity services [13, 14] to represent the capacity of the system to meet the needs of women [15]. The World Health Organization (WHO) defines a positive experience of childbirth as: “one that fulfils or exceeds a woman’s prior personal and sociocultural beliefs and expectations, including giving birth to a healthy baby in a clinically and psychologically safe environment with continuity of practical and emotional support from a birth companion(s) and kind, technically component clinical staff” [16].

Very few studies have been conducted regarding childbirth experience and related factors in Iran. A recent study in Iran [17] had demonstrated a high prevalence of negative childbirth experience among primiparous women (37%) which is higher than other countries [18, 19]. Fear of vaginal birth is one of the most significant non-medical reasons which contribute to the preference of cesarean birth among women, in turn leading to substantial prevalence of cesarean births in Iran (48%) [20]. Considering that Iranian couples are encouraged to have more children due to the decline in population and aging [21], having a high caesarian birth can jeopardize the lives of women who do not have access to health care facilities for their operational birth [6].

Childbirth in an Iranian context has been strongly influenced by medical interventions. For example, performing an episiotomy and using oxytocin during labor without women’s informed consent is a routine procedure in Iran [17]. Performing this type of intervention without the consent has been considered as obstetric violence [22]. Furthermore, most women in Iran (75%) report one or more types of disrespectful maternal care. Nearly half of women reported that they did not even have the right to move during labor and choose the delivery position [23]. The view of medicalized childbirth is a view that favors the potential power of violence and condemns woman-centered care [24]. Woman-centered care is referred to the type of care that a midwife or other care providers such as medical officer focuses on supporting woman physically, emotionally, and practically during pregnancy, childbirth, and postpartum [25]. According to the midwifery theory of Birth Territory, medicalization of childbirth can be referred as disintegrative power diminishing women’s sense of self where the health professionals do not respect women’s need and requests. The theory of Birth Territory describes, explains and predicts how a woman’s wellbeing as an embodied whole is affected by the integrative power or disintegrative power used by themselves or by others [26]. As a result of using integrative power, a woman is likely to feel good about herself, which will create satisfaction with childbirth and greater adaptation after birth and during early mothering. The midwives’ role in this theory is to support women and others to use integrative power instead of disintegrative power [24].

Although, the World Health Organization published a guideline in 2018 to improve women's childbirth experiences [16], the recommendations have not been evaluated within the Iranian childbirth context. Prior considering any of the recommendations for practice, there is a need to assess the childbirth experiences among Iranian women to identify the determinants of negative childbirth experiences with good understanding around those women’s perspectives. Considering that there was no study with accurate methodology on the childbirth experiences of Iranian women, we designed a three phased study. The first phase of the study was a quantitative where we assessed the prevalence of negative childbirth experiences and its predictors among Iranian primiparous women. In the second phase, we conducted a qualitative study with 17 in-depth- interviews aimed to understand women’s perspective on their positive or negative childbirth experiences. Finally, in order to provide effective and feasible recommendations, we conducted the Delphi study with expert panel members from different parts of the county. The present study is the first study that provides an evidence-based and culturally appropriate recommendations for a guideline to improve women’s childbirth experience within the Iranian context.

Objectives of the study:

*Phase 1:* to determine prevalence and predictors of negative childbirth experience among Iranian primiparous women.

*Phase 2:* to explore primiparous women’s perceptions regarding the determinants of negative and positive childbirth experience.

*Phase 3:* to propose evidence-based and culturally appropriate recommendations for improving of women’s childbirth experience.

## Methods

**Fig. 1:**
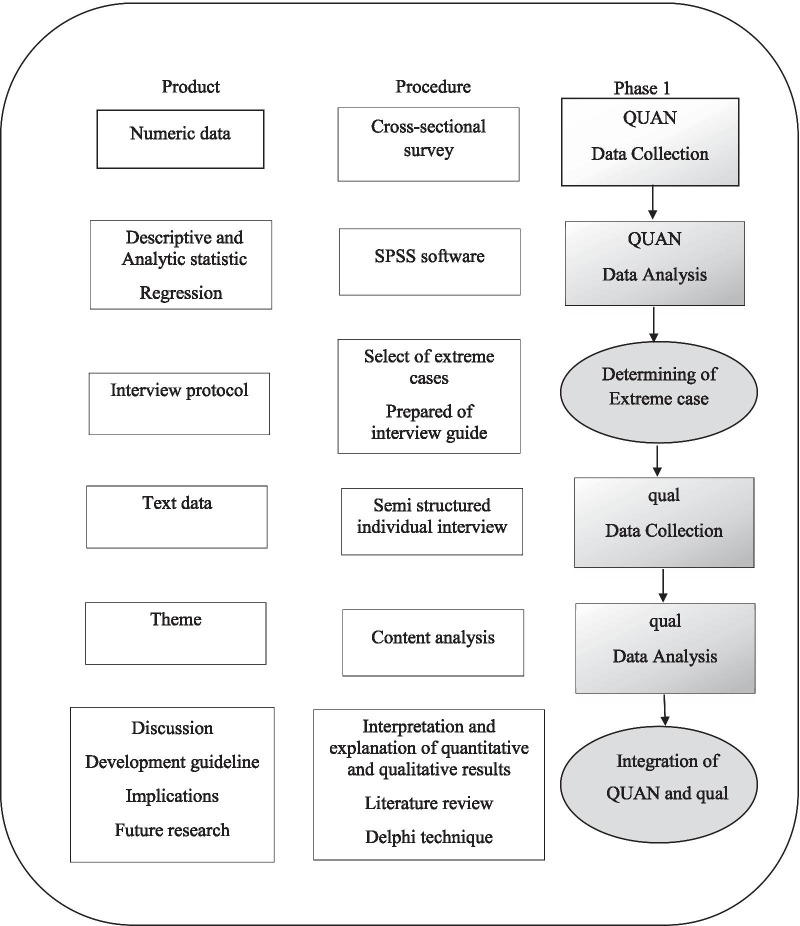
Study visual diagram

This study is a large, multiphase study aimed at developing recommendations to propose a clinical guideline for improving the Iranian primiparous women childbirth experience. The study design has already been published [27] (Fig. 1).

### First phase

#### Study design and sample size

The first phase of the study was a cross-sectional study in which the predictors of childbirth experience among primiparous women were investigated. The calculation of sample size has been reported in the protocol [27].

#### Sampling method

The sampling method was cluster sampling and 800 primiparous women at one to four months postpartum were randomly selected from multiple health centers in Tabriz. Urban health centers in Iran are mostly managed by the government. However all suburban health centers are public centers. Women’s obstetric information obtained and recorded in electronic files available in health centers. A midwife or family health expert provided women’s prenatal care. High risk women were referred to tertiary hospitals.

Tabriz urban health centers are located in different regions with different socioeconomic status. We divided the centers into three regions based on their location and socioeconomic status. Among all urban and suburban Tabriz health centers (114 centers), 64 centers were randomly selected by using the following website: www.random.org. The researcher (first author) extracted the eligible mothers’ names using electronic records and randomly selected according to quotas of each center. The mothers who were willing to participate in the study were asked to attend in the health center received extra information about the study and provide an informed consent. The exclusion criteria were women under the age of 18 years with twin, preterm or post-term, caesarian section, a history of depression, and any major neonatal abnormality.

#### Data collection tools

After obtaining informed consents, data were collected by face to face interviews. During the interview, the researcher collected the data by using the following tools: A demographic, pregnancy, labour and birth survey; Childbirth Experience Questionnaires version 2.0 (CEQ 2.0) [28, 29] and Support and Control in Birth (SCIB) [30] questionnaires. The CEQ 2.0 includes 23 items that measure the birth experience of primiparous women. Twenty items are in the form of a Likert scale (from 1 to 4 points) and 3 items in the Visual Scale (VAS) from 0 to 100. The overall score range varies from 1 to 4, and higher scores indicate a more positive birth experience [28]. The perception of support and control questionnaire (SCIB) is a 33-item questionnaire and it has been developed by Ford et al. in [30]. Each item is scored between 1 and 5. Higher scores indicate a sense of control and more support. The CEQ 2.0 and SCIB had been translated and adapted for Iranian women prior beginning of first phase. Validity and reliability of the Farsi version of CEQ 2.0 and SCIB has been verified with Cronbach's alpha of 0.94 and 0.95, respectively. The psychometric properties of the Farsi version of the CEQ 2.0 has been reported in another paper [29]. Obstetric information (length of stay in the labour room, augmentation, use of pharmacological or non-pharmaceutical methods to reduce pain, episiotomy) was obtained by referring to the hospital digital records. All of the questionnaires completed at one to four months postpartum. The quantitative phase was conducted over a period of six months.

#### Data analysis

Chi-square test was used to assess the correlation between socio-demographic, pregnancy and labour and delivery variables with birth experience status. Next, variables with p < 0.2 entered the multivariable logistic regression model with Backward strategy to determine independent covariates. Correlation between support and control in birth with childbirth experience was examined using Pearson testing. P < 0.05 was considered statistically significant.

### Second phase

#### Study design

The methodology of the second phase was qualitative content analysis with a conventional approach that explored the perception of women on the factors that related to positive and negative childbirth experiences.

#### Participants

The sampling method was purposeful and selected from the group of women who participated in the quantitative phase of the study and consented for the in-depth interview. Women were invited for the second phase of the study based on 10% of upper and lower scores in the childbirth experience scale.

#### Data collection

The qualitative phase was conducted on 17 primiparous women during the postpartum period with individual in-depth, semi-structured interviews over a period of approximately six months. The interviews were conducted in one session for each mother by the SGH (first author) and in a place where the mother felt comfortable without their support persons (mother’s house, health center and faculty room). The interviews was in Azerbaijan Turkish language. The interviewer took notes in addition to the audio recording. Non-verbal data such as facial expressions and pause were also recorded in a certain sheet. The interview protocol is available in the Additional file 1.

#### Trustworthiness

Credibility, transferability, dependability, and conformability were evaluated to ensure the accuracy of research findings [31]. Participants were selected with maximum diversity in terms of age, education, employment status and socio-economic status. The coded text was provided to four participants to confirm the accuracy of the interpretations. The data were also provided to the fifth (EM) and sixth author (MM) to ensure the consistency of the categories with the participants' statements. Their comments regarding the codes, categories and analyzes were collected in writing. The study process is described in detail to allow other researchers to examine the research process. The findings were checked by the other authors to make sure they reflect the participants' voices instead of the researcher's prejudices, motivations and views.

#### Data analysis

MAXQDA 12.13.1 was used to manage the qualitative data. The contractual content analysis was employed through the phases proposed by Graneheim and Lundmn [32]. Data analysis began by reading the whole text repeatedly to gain a complete understanding of them. Then, the texts were read word for word to extract the code. Initially, objective words were identified from the text that seemed to contain the main concepts. The researcher advanced the text by taking notes from the initial analysis, and this work continued until the preconditions for the emergence of the codes began. The codes were then categorized based on their differences or similarities. The entire coding and classification process was performed by the first author (SGH) under the supervision of the fifth author (EM), who is an expert in qualitative studies.

### Third phase

In the third phase of the study, a culture and evidence-based guideline was designed based on the findings of the qualitative and quantitative phases, a review of the literature, and the opinions of Iranian experts using the Delphi technique. This phase consisted of several steps in order to develop a document with recommendations for a proposed clinical guideline: (a) creating a structured question, (b) systematic review, (c) assessment of current evidence, and (d) Formulation of statements and their assessment (Delphi study).

#### Creating a structured question

First, a structured question was designed based on the PICO components (P = Population: Women, I = Intervention: Strategies of improving of childbirth experience, C = Comparison: Routine care or standard care, and O = Outcome = Childbirth experience): “Which interventions are appropriate with regard to policies and available facilities in Iran to improve the childbirth experience?”.

#### Systematic review

Secondly, a systematic search was done to find related guidelines and original studies from inception until September 2018 in the following databases: Cochrane Library, MEDLINE, Embase, Scopus, Web of Science, ProQuest, Magiran, SID and Barakat. The keywords were “Birth Experience”, “Childbirth Experience”, “Delivery Experience”, “Birth Satisfaction”, “Satisfaction with Birth”, “Labour Experience”, “Mother's Experience”, “Maternal Experience”, “Maternal Satisfaction”, “Birth Perception” and “Satisfaction with care”. Grey literature such as government documents from the Iranian Ministry of Health was also searched. Any randomized controlled clinical trials that reported on the effects of pharmacological and/or non-pharmacological interventions on childbirth experiences and satisfaction among women with vaginal birth were included in the systematic review. The comparison group included participants receiving standard care. The search strategy was mentioned in the Additional file 2. The selected studies were evaluated and scored by two experts in the field, using the standard tool of clinical guidelines review (AGREE = Appraisal of Guidelines Research and Evaluation). AGREE is a standard tool for reviewing clinical practice guidelines that assesses both the quality of the presentation and the quality of some aspects of the recommendations [33]. In the initial evaluation of the evidence, the RCT studies were considered to provide high evidence, while the non-random and observational studies were considered low.

#### Formulation of statements and their assessment

The results of the qualitative and quantitative phases and the findings from the literature review were combined into translated guidelines and prepared as statements for use in the Delphi technique. The aim of the Delphi was to explore the experts’ opinions regarding the recommendations that could lead to a positive or negative childbirth experience.

The Delphi technique consisted of four rounds. In the first round, experts were selected and invited to participate in the study. Informed consent was obtained from 10 experts from the fields of gynecology and obstetrician (2 experts), midwifery (2 experts) and reproductive health (7 experts) who were the academic members of different cities of Iran (Tabriz, Ardabil, Urmia, Hamadan, Ahvaz and Shiraz) (Table 1). The selection of experts was purposeful and individuals were selected based on their clinical experience with the childbirth experience concept. The experts were assured that their participation in the study was completely confidential and their comments on the entire process would be anonymized. In the second round, two open-ended questions were designed and the experts were asked to provide written responses to those questions. The questions were as follows: (1) What factors can lead to a positive experience of childbirth? (2) What factors can lead to a negative experience of childbirth? The responses were collected, coded and categorized. Duplicate responses were removed and some responses that had similar meanings were combined. The experts' responses, along with the findings of the quantitative and qualitative phases and literature review, were prepared as statements for the next round. In the third round, the experts were asked to report their own agreement or disagreement with each statement in terms of its impact on childbirth experience, feasibility, acceptability, and cost-effectiveness (e.g. provide adequate information on labour and birth during Antenatal period. Responses were rated in the Likert range from strongly agree (score 4) to strongly disagree (score 0) and not applicable. The consensus in this round was considered 70% or more. Statements in which the experts did not reach a consensus were prepared for the fourth round. In the fourth round, the experts were asked to give their final opinions concerning the remaining statements in the form of “I agree” and “I disagree.”

**Table 1:** Characteristics of experts in the Delphi technique (n = 10)

Participant	Sex	Degree (s)	Academic degree	Years of clinical experience	Area of specialty	Status of parity
1	Female	MD, Fellowship	Associate professor	24	Obstetrics, MFM^a^	Multipara
2	Female	MD, Fellowship	Professor	25	Obstetrics, MFM	Multipara
3	Female	MSc, PhD	Assistant professor	25	Health reproductive	Primipara
4	Female	MSc, PhD	Assistant professor	30	Midwifery	Multipara
5	Female	MSc	Instructor	29	Midwifery	Multipara
6	Female	MSc, PhD	Associate professor	27	Health reproductive	Multipara
7	Female	MSc, PhD	Associate professor	25	Health reproductive	Primipara
8	Female	MSc, PhD	Assistant professor	7	Health reproductive	Primipara
9	Female	MSc, PhD	Assistant professor	21	Health reproductive	Primipara
10	Female	MSc, PhD	Assistant professor	23	Health reproductive	Nullipara

^**a**^Maternal Fetal Medicine

### Ethics approval

This study has been approved by the Ethics Committee of the Tabriz University of Medical Sciences (Code: IR.TBZMED.REC.1396.786).

## Results

### First phase: quantitative phase

A total number of 800 women were enrolled in the research between 14 May 2018 and 16 November 2018 (response rate: 84%). More than half of the mothers (66.3%) were aged 18 to 25 years old with an average financial status (67.8%). Nearly half of the mothers (44.4%) had high school education and the majority of mothers (94.3%) were housewives (Table 1).

The results of the quantitative phase demonstrated that the probability of negative a childbirth experience was 4.4 times higher among women who did not have a pain relief option during labour [Odds Ratio = 4.24, 95% Confidence Interval 2.12 to 8.50; p < 0.001]. Fear of childbirth and not exercising during pregnancy increased the probability of a negative childbirth experience up to 3.47 [OR = 3.47, 95% CI 1.68 to 7.19; p = 0.001] [OR = 2.81, 95% CI 1.40 to 5.63; p = 0.003], respectively. Among the neonatal factors, skin to skin contact and lactation in the first hours after delivery had a significant relationship with the experience of childbirth (p < 0.05). Further details of the quantitative phase results are provided in previous papers [17, 34]. The results of the Pearson correlation test showed that there was statistically notable correlation between women’s sense of internal control (p < 0.001, r (standardized regression coefficient) = 0.80), external control (p = 0.001, r = 0.79) and support (p < 0.001, r = 0.83) with childbirth experience.

### Second phase: qualitative phase

After 17 interviews (8 and 9 women with negative and positive experience of childbirth, respectively), 1047 codes were extracted (519 and 528 primary codes extracted from statements of women with negative and positive childbirth experience, respectively). Forty nine subcategories were extracted for positive and negative experiences, respectively. Finally 13 categories for negative experiences and 13 categories for positive experiences were obtained which were related to the sense of internal control among women, external control and support.

Extracted categories from negative childbirth experience were as follows: (a) internal control: “Feeling of fear and worry”, and “Feeling of powerlessness”; (b) external control: “No use of pain relief methods”, “Discomfort from unnecessary interventions”, “Inappropriate physical and psychological environment of hospital”, “Maternal-neonatal complications”, “Labour dystocia” and “Not having a touch with the baby after delivery”, and “Not participating of woman and her family in care process”; and (c) support: “Disrespect and offensive behavior”, “Lack of awareness about labour and delivery”, “Unmet needs and preferences”, “Woman's hesitation due to the negative attitude of the physician and the relatives towards vaginal delivery”. Extracted categories from positive experiences were as follows: (a) internal control: “Preparedness before labour”, “Woman’s positive attitude towards vaginal delivery”, “Felling of self-control”; “Maternal and child attachment”, and “Praying and trusting in God”; (b) external control: “Woman's participation in care process”, “Short stay in the labour room”, “Satisfaction with pharmacological and non- pharmacological pain relief methods”, “Convenience and satisfaction with therapeutic interventions”, “Appropriate physical and psychological environment of hospital”, and “Woman's satisfaction with the childbirth outcomes”; and (c) support: “Receiving of family and professional support”, and “Preserving personal dignity by care provider”.

### Third phase

#### Literature review

Based on the literature review, the following international guideline was identified for supporting women to develop positive childbirth experiences during intrapartum care: “Intrapartum care for a positive childbirth experience" which was published by the World Health Organization in 2018. The WHO recommendations include 56 strategies and are divided into 6 categories [16]. A systematic review study has been conducted to identify effective interventions during labour, delivery and postpartum to create a positive perception of the childbirth experience [35]. However, given the distribution and extent of the original related studies, the research team decided to conduct and published meta-analysis studies with the aim of identifying effective interventions on childbirth experience [36, 37]. The review studies matrix is ​​presented in Table 2.

**Table 2:** Study review matrix

Authors (year)	Title and study design	Included studies	Effective interventions
Ghanbari-Homayi et al. (2019	Non pharmacological approaches to improve women’s childbirth experiences: meta-analysis	Nineteen studies	1. Encourage midwives to provide continues support to women during labour and delivery and to provide midwifery- led care 2. Providing natural interventions such as massage, hot water shower, acupressure and acupuncture
Ghanbari-Homayi et al. (2019)	Pharmacological approaches to improve women’s childbirth experiences: meta-analysis	Nine studies	1. Applying pharmacological pain relief methods (Epidural analgesia, Entonox gas, Hyoscine) to reduce labour pain
Taheri et al. (2018)	Creating a positive perception of childbirth experience	Twenty studies	1. Supporting of women during labour and delivery 2. Reduce unnecessary interventions during labour and delivery 3. Facilitating women’s preparedness before labour

### Generating a summary of recommendations using the Delphi technique

In this phase, the extracted recommendations from the literature review and the quantitative and qualitative phase were combined to generate recommendations for practice. The recommendations were divided into six categories: (a) “recommendations for pregnancy stage”, (b) “recommendations for first stage of labour”, (c) “recommendations for second stage of labour”, (d) “recommendations for third stage of labour”, (e) “recommendations for the care of newborn”, and (f) “recommendations for the care of mother after delivery”.

During technique, experts’ opinions on the factors that led to positive or negative childbirth experiences were collected and analyzed. After removing and combining duplicate responses during the first and second round of Delphi study, 37 recommendations remained and added to the other 30 recommendations that were extracted from the literature review, the quantitative and qualitative results of the study. The final 67 recommendations were assessed in the third round of the Delphi study. At this round, the experts were asked to report their own agreement or disagreement with each statement. Some statements were combined together according to the experts’ opinion. All experts reached a consensus on all 67 statements except one statement (statement number 56). The statement number 6 was extracted from WHO guidelines: “Sustained uterine massage is not recommended as an intervention to prevent postpartum hemorrhage in women who have received prophylactic oxytocin”. The reason the experts disagreed with this statement was due to the high rates of maternal mortality in Iran as a result of postpartum hemorrhages. Our recommendations were aligned with WHO recommendation and also tailored for women with Iranian culture. Our proposed recommendations for the antenatal period are novel and reported for the first time. The specific recommendations related to Iranian culture are: (1) Provide appropriate clothing for woman to keep hijab in the hospital; (2) Avoid discriminatory behavior against woman (lack of adequate care due to the sex of the fetus); (3) Provide facilities with telephones so women who have no support person during labour, birth can contact their support persons and feel safe. A lists of the main recommendations were provided in the Table 3.

**Table 3:** Final set of recommendations for improving of childbirth experience in the Iran

Statement	Consensus type	Percentage of consensus	Weighted score (%)
Recommendations for pregnancy stage			
1. Provide adequate information on labour and birth by written sources (books and the Internet) or professional health care providers during pregnancy	Completely agree	100	1.47
2. Facilitate a peer support program for pregnant woman to receive information and support from women who had previous natural childbirth experiences	Completely agree	100	1.47
3. Offer prenatal classes for all pregnant women	Completely agree	100	1.47
4. Provide information and training on the pregnancy exercises by health care professionals	Completely agree	90.9	1.44
5. Develop educational prenatal classes based on with the current care system in Iran	Completely agree	90.9	1.44
6. Facilitate situations where woman can become familiar with the labour room and birth personnel (e.g. Maternity tour or orientation session)	Completely agree	90.9	1.44
7. Provide Midwife-led continuity care models	Completely agree	100	1.47
8. Facilitate attendance and support of the woman husband or family during pregnancy	Completely agree	100	1.47
9. Facilitate identifying and consulting woman with severe fear of childbirth during pregnancy	Completely agree	90.9	1.44
Recommendations for labour, delivery and postpartum			
10. Respect the rights of pregnant woman (e.g. maintaining woman’s dignity, privacy, respectful care, the right to make informed choices, continuous s support and care with empathy and understanding during labour and delivery). Addressed to the care providers	Completely agree	90.9	1.44
11. Avoid discriminatory behavior against woman (lack of adequate care due to sex of the fetus and favoritism). Addressed to hospital staff and birth attendants	Completely agree	100	1.47
12. Establish effective communication between care providers and the woman using simple and culturally acceptable methods	Completely agree	100	1.47
13. Provide appropriate proportion ratio of care provider to woman	Completely agree	100	1.47
14. Up skill the birth centers’ staff	Completely agree	90.9	1.44
15. Provide a quiet and comfortable rooms in the emergency, labour, delivery and postpartum rooms	Completely agree	90.9	1.44
16. Provide a hygienic labour and delivery room	Completely agree	90.9	1.44
17. Provide appropriate physical space of the reception, labour and delivery room with right temperature, light, size and curtain	Completely agree	90.9	1.44
18. Provide the facilities with beds, toilets, baths and right equipment for fetal health monitoring	Completely agree	90.9	1.44
19. Provide the maternity environment with a relaxed and safe layout by using cheerful colors, pleasant smells, proper decoration and music playback	Completely agree	90.9	1.44
20. Provide appropriate and comfortable clothing for woman and personnel	Completely agree	90.9	1.44
21. Provide appropriate clothing for woman to keep hijab in the hospital	Completely agree	90.9	1.44
22. Facilitate the required conditions for the presence of an accompanying person (woman relative or husband) during labour and delivery	Completely agree	81.8	1.40
23. Provide required conditions for the presence of doula during labour and delivery	Completely agree	100	1.47
24. Provide facilities for telephone conversation for women who have no support person accompanying her during labour	Completely agree	100	1.47
25. Involve women in decision makings	Completely agree	72.7	1.29
26. Avoid unnecessary use of the bladder catheter during labour	Completely agree	100	1.47
27. Prevent fetal or neonatal complications during labour, delivery and postpartum	Completely agree	100	1.47
28. Mandate the auscultation using Doppler ultrasound device or Pinard fetal stethoscope for the assessment of fetal health on labour admission	Completely agree	100	1.47
29. Limit unnecessary labour admissions, such as admitting a healthy women presenting within latent phase of labour	Completely agree	72.7	1.29
30. Provide sufficient information on the definitions of the latent and active phase of labour and duration of labour	Completely agree	72.7	1.29
31. Reinforce adequate information about care process or interventions in the woman’s own language	Completely agree	100	1.47
32. Keep the woman informed about her condition, her fetus and baby during labour and birth	Completely agree	72.7	1.29
33. Avoid unnecessary vaginal examinations to evaluate the routine active phase of labour in low-risk woman	Completely agree	72.7	1.29
Recommendations for first stage of labour			
34. Reassure intermittent auscultation of the fetal heart rate with either a Doppler ultrasound or Pinard fetal stethoscope for healthy women in labour”	Completely agree	100	1.47
35. Facilitate providing Epidural analgesia for healthy pregnant women who request epidural pain relief during labour	Completely agree/agree	72.7	1.29
36. Facilitate providing parenteral opioids, such as fentanyl, diamorphine and pethidine, for healthy pregnant women who request pain relief during labour, depending on her preferences”	Completely agree/agree	90.9	1.44
37. Facilitate relaxation techniques, including progressive muscle relaxation, breathing, music, mindfulness and other techniques, for healthy pregnant women who request pain relief during labour, depending on a her preferences	Completely agree	100	1.47
38. Facilitate providing manual pain relief techniques, such as massage or application of packs, for healthy pregnant women requesting pain relief during labour, depending on a woman’s preferences”	Completely agree	100	1.47
38. Keep the low risk woman hydrated by offering oral fluid and food intake during labour	Completely agree	100	1.47
40. Encourage women at low risk, to move and change position during labour	Completely agree	100	1.47
41. Avoid routine amniotomy, early oxytocin administration, antispasmodics, and intravenous fluids	Completely agree	88.9	1.41
42. “Avoid routine clinical pelvimetry on admission in labour for healthy pregnant women”	Completely agree	100	1.47
43. Avoid routine cardiotocography for the assessment of fetal well-being on labour admission of healthy pregnant women presenting with spontaneous labour	Completely agree/agree	72.7	1.41
44. Avoid routine perineal/ pubic shaving prior to giving vaginal birth	Completely agree	100	1.47
45. Avoid performing enema for reducing the use of labour augmentation	Completely agree	100	1.47
46. “Avoid routine vaginal cleaning with chlorhexidine durng larour for the purpose of preventing infections morbidities as it is not recommended”	Completely agree	100	1.47
Recommendations for second stage of labour			
47. Provide information on the definition and duration of the second stage of labour for women	Completely agree	72.7	1.29
48. “Encourage and support women in the exclusive phase of the second stage of labour s to follow their own urge to push”	Completely agree	90.9	1.44
49. Avoid manual fundal pressure during the second phase of labour	Completely agree	100	1.47
50. Avoid routine use of episiotomy for women undergoing spontaneous labour	Completely agree	90.9	1.44
51. Allow the woman to choose her birth position during delivery	Completely agree	72.7	1.29
Recommendations for third stage of labour			
52. Offer routine use of uterotonics for the prevention of postpartum haemorrhage (PPH) during the third stage of labour	Completely agree	90.9	1.44
53. “Delaye umbilical cord clamping (not earlier than 1 min after birth”	Completely agree	90.9	1.44
54. Provide controlled cord traction (CCT) for vaginal births (if the care provider and the parturient woman regard a small reduction in blood loss and a small reduction in the duration of the third stage of labour as important)	Completely agree/ agree	81.8	1.27
55. Provide adequate anesthesia during episiotomy and its repair	Completely agree	100	1.47
56. Avoid a sustained uterine massage as an intervention to prevent postpartum haemorrhage in women who have received prophylactic oxytocin	Completely agree/ agree	63.6	1.10
Recommendations for care of the newborn			
57. Avoid routine suctioning of the mouth and nose for neonates who start breathing on their own after birth	Completely agree	100	1.47
58. Facilitate skin-to-skin contact with the mother for the Newborn who have with complications to prevent hypothermia and promote breastfeeding”	Completely agree	100	1.47
59. Facilitate breastfeeding initiation in first hour after birth for all newborns stable when the mother and baby are ready, including low-birth-weight (LBW)	Completely agree	100	1.47
60. Do not separate the mother and baby without any medical reason. Keep them in the same room during day and night	Completely agree	100	1.47
61. Administrate 1 mg of vitamin K intramuscularly with parents’ consent	Completely agree	100	1.47
Recommendations for care of the mother after delivery			
62. “Assess uterine tons for early identification of uterine atony for all women”	Completely agree	100	1.47
63. Routinely assess postpartum women for vaginal bleeding, uterine contraction, fundal height, temperature and heart rate (pulse) during the first 24 h starting from the first hour after birth	Completely agree	100	1.47
64. Provide maternity and neonatal care at least for 24 h after an uncomplicated vaginal birth in a health care facility	Completely agree	81.8	1.40
65. Avoid administrating routine antibiotic prophylaxis for women with uncomplicated vaginal birth	Completely agree	81.8	1.40
66. “Avoid routine antibiotic prophylaxis for women with episiotomy”	Completely agree	81.8	1.40
67. Be responsive to a woman's complications at any time of pregnancy, delivery and postpartum. Addressed to all health care providers	Completely agree	81.8	1.40

## Discussion

Based on this three-phased study, 66 recommendations were developed to be considered in a clinical guideline towards improving Iranian childbirth experiences among women with a vaginal birth. The proposed guideline is unique, evidence-based and woman-centred as it is based on the Iranian women’s childbirth experiences, a systematic literature review, WHO guideline, and the input from a diverse Iranian clinical experts.

The primary recommendations include: (a) providing antenatal education for all women to learn more about pregnancy how to look after themselves during antenatal, birth and postpartum periods; and (b) providing a safe, non-discriminatory and woman-centred environment where women have opportunity to receive midwifery care, use pain relief and benefit from appropriate equipment and expert’s input towards minimizing any adverse maternal and neonatal outcome.

The findings of the quantitative and qualitative phases of the study showed that the woman-centered approach in the Iranian maternity services has been neglected and the rights of pregnant women are not well-respected. In the woman-centered approach, the focus of the care is on the woman's personal needs, expectations and desires. This approach emphasises the relationship between women and midwives during pregnancy and childbirth, and addresses the concept of empowering women which is the priority of midwifery-led care [24, 25]. In order to protect the rights of pregnant women, the attitude of society and care providers towards the gender of women and the issue of pregnancy and childbirth need to be systematically adjusted.

Although Iran's health care system has been working to reduce the mortality and morbidity of pregnant women [38]. Woman’s satisfaction with the childbirth experience is one of the main indicators of quality of care. The care is considered poor if a woman is not satisfied with her experienced [39]. Therefore, by medicalization of the childbirth, intangible consequences such as the psychological effects of childbirth should not be neglected. Evidence has demonstrated a high maternal satisfaction with performing midwifery-led care where women feel that there are increased opportunities to be heard and cared for [40].

Many of the recommendations from our study were consistent with WHO recommendations, however, there are extra recommendation that were tailored for women in Iran. According to the findings of the qualitative phase of the study, some participants complained that hospitals did not provide appropriate support for women to maintain their hijab. Majority of experts (90.9%) stated the necessary of maintaining hijab for women who wish to keep their hijab during labour and birth. Women also reported that they were ashamed and embarrassed that male caregivers or workers had seen or physically visited the examination or labor room. Other specific issue during labor and birth in the most Iranian hospitals was that women’s support people were not allowed to company the women during labour and birth caused by having a shared labour room among several women. Therefore, the only option for women is to ask for a phone call to their spouse or support people. Additionally, women in the public hospitals are discriminated based on the fetus’s gender or being a stranger in the hospital (without having any personal relationship or connections to the health professionals and staff in the hospital). Another element is the lack of resources and pain relief options (non-pharmacological and pharmacological) for women during labour and birth. Many of obstetricians and midwives in Iran still do not have a positive attitude towards the use of pain relief during labour and birth. The present study has also added extra recommendations for antenatal care. For example, the finding of our study shown that exercising during pregnancy was associated with positive childbirth experience among primiparus women. Antenatal recommendations along with other recommendations are culturally safe for Iranian women.

Considering that the Iranian health system is mainly focused on reducing the morbidity and mortality of women and infants, our recommendations that relate to the mother’s physical health, fetuses and infants can be easily adopted and implemented. However, the lack of adequate awareness of health care providers about the consequences of women’s negative childbirth experiences is a serious obstacle in the implementation of the following recommendations: respectful maternal care, establishing an effective relationship between care provider and women, providing sufficient antenatal education about labor, birth and postpartum care, responding to women’s need and concerns, involving women in decision making, reduction of unnecessary interventions, and offering pain relief options.

## Strengths and limitations

One of the strengths of the study is the inclusion of women living in suburban areas in addition to urban from both private hospitals and public hospitals. High sample size, high responsiveness (84%), random selection and the use of standard and validated tools for Iranian women to evaluate women's childbirth experiences are the strengths of the quantitative phase of the study. Response bias could be as a potential sources of bias in the study. To prevent this type of bias, all of the questionnaires were completed by the first author at least one month after birth when women could attend health care centers without being concerned about their birth care providers.

Purposeful selection of women in the qualitative phase that included 10% of women with high or low score in childbirth experience scale is another strength because we were able to use the childbirth experiences of both spectrums to formulate recommendations.

Although in practice we could not include women living in other cities of Iran, we used the views of experts in major cities of Iran (in terms of ethnic diversity and geographical location) in the Delphi phase. Still, the recommendations should be cautiously considered for cities and ethnicities. Considering that the results of the study is limited to low risk, adult, primiparous women with term singleton healthy newborns, the recommendations of the guideline cannot not be generalized to all groups of women such as high risk women, multiparous, women with caesarian birth.

### Implication for practice

The findings of the study can be applied in various educational, managerial, and clinical areas. The findings of this study can be used to develop the evidence based content and design appropriate teaching methods in prenatal classes for women.

The results of the present study can also be incorporated into the tertiary education of midwives, doctors, nurses and other health care providers to alter or improve their attitude and practice towards the sensitive issue of childbirth experience. Normalizing the protection of women's rights in maternity care services, can raise the level of awareness of men and women in society to respect women during childbirth who are vulnerable and at risk of a negative childbirth experience.

The findings of this study can also be considered in policy making and management of health centers. For instance, providing antenatal consultation time and continuous support for women who are extremely afraid of vaginal birth can be effective approach in preventing the negative experience of childbirth. Adopting policies to increase non-pharmacological pain relief for women and providing pharmacological pain options are useful strategies to enhance women’s satisfaction during labor and birth. Policies in maternity services should highlight the importance of skin to skin contact for both women and health care professionals; and facilitate implementation of skin to skin contact after birth for women and their infants.

Enhancing positive childbirth experience fits well with the priorities of the Ministry of Health and Medical Education in Iran towards reduction of non-medical reasons for cesarean section births [20]. According to the findings of the qualitative phase of the study, it is necessary to increase women's awareness about their choices and rights to involve in decision making process around obstetric interventions. Additionally, maternity service managers need to consider new practices to welcome the presence of the family members including women’s husband and family during labor m birth and early postpartum. Caregivers need to be educated to provide a friendly, respectful and emotionally safe environment for women where they can focus on women’s needs, values and preferences. Identifying the existing barriers and enablers for implementing the recommendations can pave the way for further research.

## Conclusion

A guideline was developed with 66 recommendations for pregnancy, labour, delivery and postpartum stages to improve the childbirth experience of Iranian primiparous women. This is first evidence-based guideline in which has been designed for the Iranian culture. Given the high prevalence of negative childbirth experience among Iranian primiparous women, it seems that the present study may be of great interest for managers, leaders, policymakers, and care providers. However, further studies are required to identify the enablers, barriers and to assess the effectiveness of the guideline.

## Supplementary Information

**Additional file 1. ** Interview Guide Questions.

**Additional file 2.** Search strategy.

## Data Availability

Not applicable.

## Abbreviations

WHO: World Health Organization
CEQ 2.0: Childbirth Experience Questionnaire 2.0
SCIB: Support and Control in Birth
