# Supplementary material for: Recommendations for improving primiparous women’s childbirth experience: results from a multiphase study in Iran
Source: Reprod Health. 2021 Jul 6;18:146. doi: 10.1186/s12978-021-01196-7 (PMC8259137; doi:10.1186/s12978-021-01196-7)
Supplement: Supplementary file 1 — Additional file 1. Interview Guide Questions. [file 12978_2021_1196_MOESM1_ESM.docx]

**Interview Guide Questions**

|  | **Question** |
| --- | --- |
| **1** | Please explain about the recent childbirth and your childbirth experience. |
| **2** | What factors make you feel good and satisfied during your childbirth? |
| **3** | What factors make you feel bad and dissatisfied during your childbirth? |
| **4** | Who has supported you and how has it been effective for you in this experience? |
