# Supplementary material for: Recommendations for improving primiparous women’s childbirth experience: results from a multiphase study in Iran
Source: Reprod Health. 2021 Jul 6;18:146. doi: 10.1186/s12978-021-01196-7 (PMC8259137; doi:10.1186/s12978-021-01196-7)
Supplement: Supplementary file 2 — Additional file 2. Search strategy. [file 12978_2021_1196_MOESM2_ESM.docx]

**Search strategy**

| **Database** | **Key words’ combination** |
| --- | --- |
| **Web of Sciences** | (“birth experience*” OR “experience of birth*” OR “childbirth experience*” OR “experience of childbirth*” OR “delivery experience*” OR “experience of delivery*” OR “labour experience*” OR “experience of labour*” OR “mother’s experience*” OR “experience of mother*” OR “maternal experience*” OR “birth satisfaction*”, “satisfaction with birth*” OR “childbirth satisfaction*” OR “satisfaction with childbirth*” OR “birth perception*” OR “perception of birth*” OR “childbirth perception*” OR “perception of childbirth*” OR “satisfaction with care”) AND (trial* OR RCT* OR intervention* OR prevention*) |
| **Embase**  **Medline**  **Scopus** | #1 “birth experience*”.mp. OR “experience of birth*”.mp.  #2 “childbirth experience*”.mp. OR “experience of childbirth*”.mp.  #3 “delivery experience*”.mp. OR “experience of delivery*”.mp.  #4 “labour experience*”.mp. OR “experience of labour*”.mp.  #5 “mother’s experience*” mp. OR “experience of mother*”.mp.  #6 “maternal experience*”.mp.  #7 “birth satisfaction*”.mp. “satisfaction with birth*”.mp. OR “childbirth satisfaction”.mp. OR “satisfaction with childbirth” .mp.  # 8 “birth perception*”.mp. OR “perception of birth*”.mp. OR “childbirth perception*”.mp. OR “perception of childbirth*”.mp.  #9 “maternal satisfaction*”.mp.  #10 “satisfaction with care” .mp.  #11 OR/#1 - #10  #12 trial*.mp.  #13 RCT*.mp.  #14 intervention*.mp.  #15 prevention*.mp.  #16 OR/#12 - #16  #17 AND/#11, #16 |
